# Application of the ARIMA model on the COVID-2019 epidemic dataset

**DOI:** 10.1016/j.dib.2020.105340

**Published:** 2020-02-26

**Authors:** Domenico Benvenuto, Marta Giovanetti, Lazzaro Vassallo, Silvia Angeletti, Massimo Ciccozzi

**Affiliations:** a Unit of Medical Statistics and Molecular Epidemiology, University Campus Bio-Medico of Rome, Italy; b Laboratório de Flavivírus, Instituto Oswaldo Cruz, Fundação Oswaldo Cruz, Rio de Janeiro, Brazil; c Department of Financial and Statistical Sciences, University of Salerno, Salerno, Italy; d Unit of Clinical Laboratory Science, University Campus Bio-Medico of Rome, Italy

**Keywords:** COVID-2019 epidemic, ARIMA model, Forecast, Infection control

## Abstract

Coronavirus disease 2019 (COVID-2019) has been recognized as a global threat, and several studies are being conducted using various mathematical models to predict the probable evolution of this epidemic. These mathematical models based on various factors and analyses are subject to potential bias. Here, we propose a simple econometric model that could be useful to predict the spread of COVID-2019. We performed Auto Regressive Integrated Moving Average (ARIMA) model prediction on the Johns Hopkins epidemiological data to predict the epidemiological trend of the prevalence and incidence of COVID-2019. For further comparison or for future perspective, case definition and data collection have to be maintained in real time.

**Table:** Specifications Table

Subject	Infectious Diseases
Specific subject area	Econometric models applied to infectious diseases epidemiological data to forecast the prevalence and incidence of COVID-2019
Type of data	Chart Graph Figure
How data were acquired	Gretl 2019d http://gretl.sourceforge.net/win32/index_it.html
Data format	Data are in raw format and have been analyzed. An Excel file with data has been uploaded.
Parameters for data collection	Parameters used for ARIMA were model ARIMA (1,2,0) and ARIMA (1,0,4)
Description of data collection	The daily prevalence data of COVID-2019 from January 20, 2020 to February 10, 2020 were collected from the official website of Johns Hopkins university (https://gisanddata.maps.arcgis.com/apps/opsdashboard/index.html), and Excel 2019 was used to build a time-series database. Descriptive analysis of the data was performed, and to evaluate the incidence of new confirmed cases of COVID-2019 and to prevent eventual bias, the difference between the cases confirmed on that day and the cases confirmed on the previous day were calculated Δ(X_n_-X_n-1_).
Data source location	University Campus Bio-Medico of Rome
Data accessibility	Raw data can be retrieved from the Github repository https://github.com/CSSEGISandData/COVID-19

**Table:** 

**Value of the Data** • These data are useful because they provide a forecast for COVID-2019 epidemic, thus representing a valid and objective tool for monitoring infection control. • All institutions involved in public health and infection control can benefit from these data because by using this model, they can daily construct a reliable forecast for COVID-2019 epidemic. • The additional value of these data lies in their easy collection and in the possibility to provide valid forecast for COVID-2019 daily monitoring after the application of the ARIMA model. • These data represent an easy way to evaluate the transmission dynamics of COVID-2019 to verify whether the strategy plan for infection control or quarantine is efficient.

## 1 Data description

**Fig. 1:**
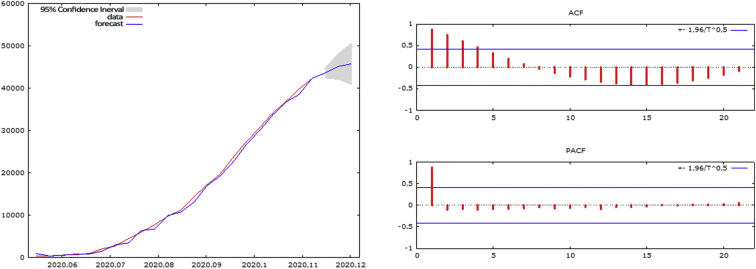
Correlogram and ARIMA forecast graph for the 2019-nCoV prevalence.

The daily prevalence data of COVID-2019 from January 20, 2020 to February 10, 2020 were collected from the official website of Johns Hopkins University (https://gisanddata.maps.arcgis.com/apps/opsdashboard/index.html), and Excel 2019 was used to build a time-series database [1]. ARIMA model was applied to a dataset consisting of 22 number determinations. Fig. 1 shows that the overall prevalence of COVID-2019 presented an increasing trend that is reaching the epidemic plateau. The difference between cases of one day and cases of the previous day Δ(Xn-Xn-1) showed a nonconstant increase in the number of confirmed cases. Descriptive analysis of the data was performed to evaluate the incidence of new confirmed cases of COVID-2019 and to prevent eventual bias.

## 2 Experimental design, materials, and methods

The ARIMA model includes autoregressive (AR) model, moving average (MA) model, and seasonal autoregressive integrated moving average (SARIMA) model [2]. The Augmented Dickey-Fuller (ADF) [3] unit-root test helps in estimating whether the time series is stationary. Log transformation and differences are the preferred approaches to stabilize the time series [4]. Seasonal and nonseasonal differences were used to stabilize the term trend and periodicity.

Parameters of the ARIMA model were estimated by autocorrelation function (ACF) graph and partial autocorrelation (PACF) correlogram. To determine the prevalence of COVID-2019, ARIMA (1,0,4) was selected as the best ARIMA model, while ARIMA (1,0,3) was selected as the best ARIMA model for determining the incidence of COVID-2019. Gretl2019d statistical software [5] was used to perform statistical analysis on the prevalence and incidence datasets, and the statistical significance level was set at 0.05. A previous study was considered as reference for the methodology of the analysis [6].

Logarithmic transformation was performed to evaluate the influence of seasonality on the forecast. The correlogram reporting the ACF and PACF showed that both prevalence and incidence of COVID-2019 are not influenced by the seasonality. The forecast of prevalence and incidence data with relative 95% confidence intervals are reported in Table 1.

**Table 1:** Forecast value for the 2 days after the analysis for the prevalence and for the incidence of the COVID-2019.

	Date	Forecast	95% Confidence Interval
Prevalence	11/02/2020	43599.71	42347.53–44851.9
	12/02/2020	45151.45	42084.88–48218.02
Incidence	11/02/2020	2070.66	1305.23–2836.09
	12/02/2020	2418.47	1534.43–3302.51

**Fig. 2:**
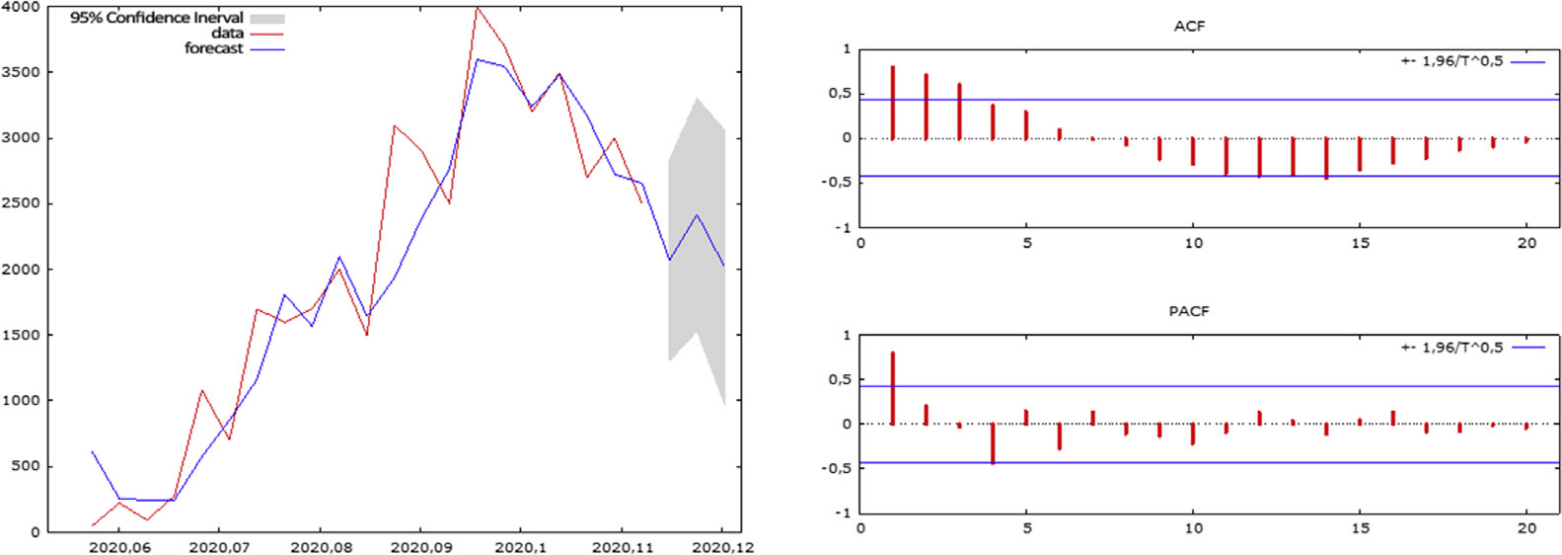
Correlogram and ARIMA forecast graph for the 2019-nCoV incidence.

Although more data are needed to have a more detailed prevision, the spread of the virus seems to be slightly decreasing. Moreover, although the number of confirmed cases is still increasing, the incidence is slightly decreasing. If the virus does not develop new mutations, the number of cases should reach a plateau (Fig. 1, Fig. 2). The forecast and the estimate obtained are influenced by the “case” definition and the modality of data collection. For further comparison or for future perspective, case definition and data collection must be maintained in real time.

## References

[1] (2019). Johns Hopkins University Center for Systems Science and Engineering.

[2] Fattah J., Ezzine L., Aman Z., El Moussami H., Lachhab A. (2018). Forecasting of demand using ARIMA model. Int. J. Eng. Bus. Manag..

[3] Cao S., Wang F., Tam W., Tse L.A., Kim J.H., Liu J., Lu Z. (2013). A hybrid seasonal prediction model for tuberculosis incidence in China. BMC Med. Inf. Decis. Making.

[4] Cheung Y.-W., Lai K.S. (1995). Lag order and critical values of the augmented Dickey–Fuller test. J. Bus. Econ. Stat..

[5] Baiocchi G., Distaso W. (2003). GRETL: econometric software for the GNU generation. J. Appl. Econom..

[6] Wang Y.W., Shen Z.Z., Jiang Y. (2018). Comparison of ARIMA and GM(1,1) models for prediction of hepatitis B in China. PloS One.

